# Bioactive Compounds and Antimicrobial Activities in Iranian *Crataegus persica* Ecotypes for Potential Food and Medicinal Uses

**DOI:** 10.1002/fsn3.4748

**Published:** 2025-01-09

**Authors:** Ghasem Eghlima, Fateme Aghamir, Meisam Mohammadi, Hanifeh Seyed Hajizadeh, Ozkan Kaya

**Affiliations:** ^1^ Department of Agriculture Medicinal Plants and Drugs Research Institute, Shahid Beheshti University Tehran Iran; ^2^ Department of Horticulture, Faculty of Agriculture Ilam University Ilam Iran; ^3^ Department of Horticulture, Faculty of Agriculture University of Maragheh Maragheh Iran; ^4^ Republic of Turkey Ministry of Agriculture and Forestry Erzincan Horticultural Research Institute Erzincan Turkey; ^5^ Department of Life Sciences Western Caspian University Baku Azerbaijan

**Keywords:** fruit morphology, nutritional value, phytochemical, pigments, Rosaceae

## Abstract

The genus *Crataegus*, belonging to the Rosaceae family, exhibits widespread distribution across Iran, comprising 17 species. Hawthorn has garnered significant attention in recent years as a prominent herbal remedy in phytotherapy and culinary applications. Various plant parts, including flowers, leaves, and fruits, have been traditionally employed to address cardiovascular conditions such as hypertension, hypotension, palpitations, and cardiac arrhythmias. The presence of bioactive phytochemicals, notably polyphenols and anthocyanins, confers antispasmodic and analgesic properties to hawthorn. This study investigated the bioactive capacity of mature fruits from five *Crataegus persica* ecotypes indigenous to Iran. High‐performance liquid chromatography (HPLC) was utilized to elucidate the phenolic profile, although morphological characteristics and antimicrobial properties were also assessed. The ecotypes under investigation were sourced from elevations ranging from 1205 to 1681 m above sea level. Morphological analysis revealed that fruit weight varied from 0.84 to 2.53 g, whereas pericarp weight ranged from 0.21 to 1.19 g. The number of seeds per fruit fluctuated between 1 and 3. HPLC analysis identified the primary phenolic compounds in *C. persica* as catechin, *p*‐coumaric acid, quercetin, chlorogenic acid, and rutin. Biochemical characterization of the ecotypes yielded the following ranges: total soluble solids (TSS) 10.31°Brix–18.53°Brix, total soluble carbohydrates (TSC) 8.34%–16.65%, vitamin C content 4.24–12.38 mg g^−1^ DW, total carotenoid content 185.32–294.4 μg g^−1^ DW, and total anthocyanin content 43.21–85.34 mg C3G g^−1^ DW. Total phenolic compounds ranged from 170.52 to 254.31 mg GAE g^−1^ DW, whereas total flavonoid content varied between 59.28 and 84.41 mg RE g^−1^ DW. Antioxidant activity, as determined by IC_50_ values, ranged from 14.47 to 37.51 μg mL^−1^. Antimicrobial assays demonstrated that the *C. persica* extract exhibited maximum efficacy against *Staphylococcus aureus* , with the *CP3* ecotype extract displaying a minimum inhibitory concentration (MIC) of 0.125 mg mL^−1^. In conclusion, this comprehensive analysis of five distinct *C. persica* ecotypes (*CP1*, *CP2*, *CP3*, *CP4*, and *CP5*) revealed substantial diversity in terms of morphological traits, functional bioactive compounds, and antimicrobial potential. These findings contribute to the growing body of knowledge regarding the phytochemical composition and potential therapeutic applications of *Crataegus persica*.

## Graphical abstract

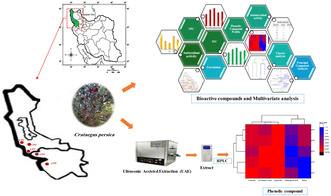

## 1 Introduction

The utilization of medicinal plants as novel therapeutic agents has garnered significant attention in recent years, primarily because of the presence of bioactive compounds that exhibit potential efficacy in treating various ailments. Notably, wild species often contain higher concentrations of antioxidants, which are crucial for human health, compared to their cultivated counterparts (Li et al. 2016). In this regards, hawthorn (*Crataegus* spp.), a member of the Rosaceae family, is a fruit‐bearing plant that grows naturally in Iran. Its distribution predominantly spans the temperate regions of the world, typically between 30° and 50° N latitude (Phipps 1983), encompassing northern areas of East Asia, Europe, and North America (González‐Jiménez et al. 2018). The fruit of this plant has been associated with numerous beneficial effects on human health. Hawthorn, characterized by its thorny nature, thrives primarily on limestone soils in wooded and sunny areas up to 1300 m above sea level in Iran. As a prominent tree of the Zagros Mountains, it is widely distributed across Iran, Syria, Palestine, Lebanon, Iraq, Anatolia, and Turkmenistan (Sagheb Talebi et al. 2014). The genus *Crataegus* comprises over 1000 species, including hybridized varieties (Edwards et al. 2012), with approximately 22 species identified in Iran (Alirezalu et al. 2018). The therapeutic efficacy of *Crataegus* flower and fruit extracts can be attributed to beneficial components such as epicatechin, hyperoside, and chlorogenic acid, which exhibit free radical scavenging properties and are among the most potent antilipoperoxidants (Rakotoarison et al. 1997). However, only a select few species within this genus are utilized for medicinal purposes.

The medicinal properties of hawthorn species are primarily associated with their polyphenolic content. The plant extract is rich in flavonoids and hydroxycinnamic acids, with most compounds extracted from leaves and flowers standardized on the basis of total flavonoid content (Ding et al. 2010). The type and percentage of flavonoids are considered key indicators of plant quality (Urbonavičiūtė et al. 2006). Flavonoids, more than other secondary metabolites, are extensively employed in chemotaxonomic studies because of their distinct quantitative and qualitative patterns (Ringl et al. 2007), abundance, stability, and ease of detection (Švehlíková et al. 2002). Extant literature suggests that the phenolic composition of *C. persica* fruit differs significantly from other species and ecotypes within the genus. A comparative study of various *Crataegus* species, including *C. monogyna* , *C. meyeri* , *C. pseudoheterophylla*, *C. pentagyna* , and *C. pontica* , revealed that *C. meyeri* , *C. pentagyna* , and *C. pseudoheterophylla* contained notably high percentages of phenolic, tannic, and flavonoid compounds (Alirezalu et al. 2020). These findings indicate the potential of phytochemical profiling as a viable approach for identifying species with medicinal priority among different genera and ecotypes of medicinal plants. The therapeutic potential of *Crataegus* spp. is primarily attributed to their antioxidant content, which is associated with phytochemicals such as ascorbic acid, carotenoids, and flavonoid compounds (Donno et al. 2017). Recent HPLC‐DAD analyses have detected the presence of eight phenolic compounds in *C. monogyna* extract and nine compounds in *C. laciniata* extract, with coumaric acid uniquely present in the latter. Both extracts exhibited high concentrations of polyphenols, flavonoids, and condensed tannins. Furthermore, all three *Crataegus* species tested demonstrated significant antioxidant activity against 2,2‐diphenyl‐1‐picrylhydrazyl (DPPH) and in the ferric reducing antioxidant power (FRAP) assay (Radi et al. 2023). The quality parameters of *Crataegus* fruits include fruit acids, which facilitate nutrient digestion and stimulate blood circulation. These acids are rapidly oxidized in the body and do not exert harmful effects. Moreover, their salts are alkaline‐forming elements, rendering them highly important in human nutrition. *Crataegus* fruits also contain substantial amounts of essential minerals such as Ca, K, Mg, Na, and P (Özcan et al. 2005).

Wild species of edible fruits exhibit considerable diversity in morphology, fruit quality, performance, and nutrient content compared to their domesticated counterparts. These traits can be significantly influenced by environmental conditions and ecotype variations (Gundogdu et al. 2014). Consequently, ecotype selection is a critical initial step in the development of appropriate varieties. Therefore, comprehensive studies on *Crataegus* ecotypes, focusing on important nutritional traits and phytochemical content, are of paramount importance. Although the antioxidant activity of hawthorn fruits has been extensively reported in the literature (Ruiz‐Rodríguez et al. 2014; Ding et al. 2010; Alirezalu et al. 2020) *C. persica* remains a prominent species in Iran, traditionally used for medicinal purposes. Given its endemic status in Iran and the paucity of comprehensive phytochemical information on its various ecotypes, the present study aims to: (I) investigate the profile of bioactive compounds in *C. persica* fruit extracts using high‐performance liquid chromatography (HPLC); (II) examine the morphological characteristics *of C. persica* fruits; (III) evaluate the antimicrobial activity of *C. persica* fruit extracts; and (IV) identify elite ecotypes for potential commercial development of *C. persica* .

## 2 Materials and Methods

### 2.1 Plant Materials

**FIGURE 1:**
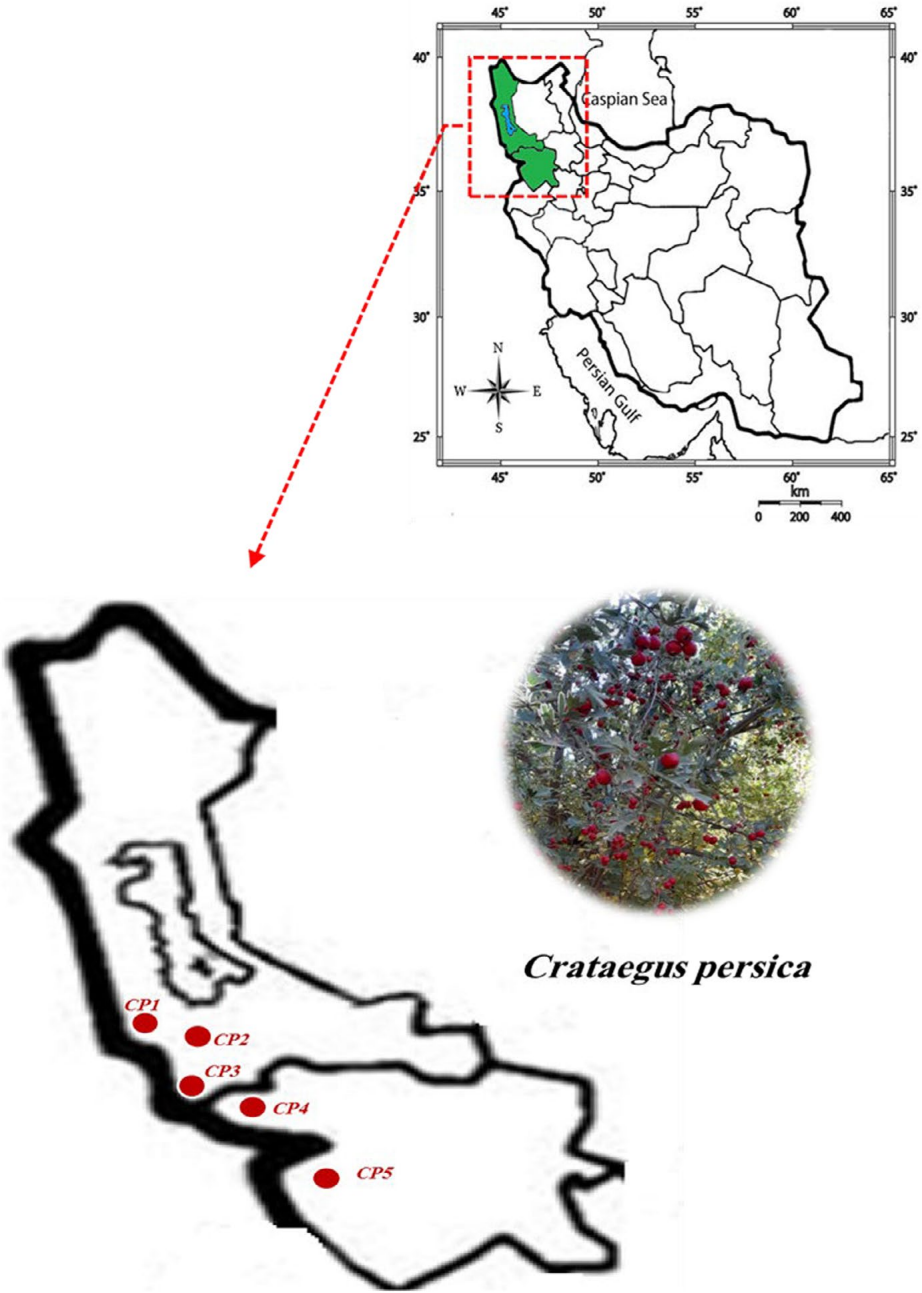
Distribution map of the 14 populations of *Crataegus persica* collected across Iran.

Ripe fruits of five *C. persica* ecotypes were collected from two provinces of Iran in the fall season of 2023. Ten individuals were selected from each ecotype at 200 m. The collected samples were identified by Prof. Ali Sonboli, and voucher specimens were deposited at the Shahid Beheshti University herbarium. The climatic and geographical characteristics of the place where the samples were collected are shown in Figure 1 and Table 1.

**TABLE 1:** Geographical description for the collection sites of *Crataegus persica* ecotypes.

No.	Location	Code	Latitude (N)	Longitude (E)	Altitude (m)	MAT (°C)	MAP (mm)
1	West Azerbaijan‐Piranshahr	*CP1*	36°44′	45°56′	1681	12.0	341
2	West Azerbaijan‐Mahabad	*CP2*	36°30′	45°33′	1485	11.5	368
3	West Azerbaijan‐Sardasht	*CP3*	36°26′	45°55′	1205	13.5	870
4	Kurdistan‐Baneh	*CP4*	35°58′	45°49′	1526	14.0	684
5	Kurdistan‐Marivan	*CP5*	35°32′	46°16′	1482	14.2	835

Abbreviations: MAP, means annual precipitation; MAT, means annual temperature.

### 2.2 Morphological Attributes Analysis

To evaluate the morphological traits and fruit quality, about 30 fruits were randomly selected from each individual and the traits were evaluated, and the average was recorded as representative of that individual. Fruit length and width, fruit fresh weight, pericarp weight, seed weight, and number of seeds per fruit were measured. A digital caliper was used to measure the length and width of the fruit, and the characteristics of fresh and dry weight of the fruit and pericarp weight were determined by a digital scale.

### 2.3 Extraction and Analysis of Phenolic Compounds by HPLC

To extract, 200 mg of fruit pericarp powder was extracted in 10 mL of 80% methanol using ultrasonic waves produced by an ultrasonic device (SingenHtw Elmasonic‐D 78224—Elma Germany) for 20 min at a temperature of 40°C (Bakhtiar et al. 2023). Then, using a centrifuge, it was centrifuged for 10 min at a speed of 4000 rpm and kept in a refrigerator at a temperature of −18°C until the time of analysis. Identification and measurement of phenolic compounds were done with an HPLC system manufactured by Knauer (Germany) equipped with two pumps (model—Wellchron‐K1001) and a PDA detector (model DP‐M20A). The column used was an RP‐C18 type with a 4.6 mm inner diameter and a 250 mm length (manufactured by Eurosphr). The mobile phase consisted of methanol, water, and trifluoroacetic acid solvents (90:10:0.02 v/v/v) and was run at a rate of 0.5 mL min^−1^. Pure standard phenolic compounds were obtained from Sigma Aldric company, and various concentrations (5, 10, 20, 40, 60, 80, 100, 150, and 200 ppm) of standard compounds were prepared to create calibration curves for analysis. Only a limited number of phenolic compounds were utilized. The quantity of phenolic compounds was ultimately expressed as μg g^−1^ DW of the plant.

### 2.4 Total Soluble Solids and Total Soluble Carbohydrate Assay

The total soluble solids (TSS), in the fruits were assessed using a handheld refractometer (model 9703, Japan). Additionally, the total soluble carbohydrate content (TSC) of the fruit samples was determined using the anthrone method (Cao et al. 2008).

### 2.5 Vitamin C Assay

The amount of vitamin C was determined using the AOAC method (AOAC 2014) with ascorbic acid standard. 1 mL of metaphosphoric acid (3%) was mixed with 1 g of fruit pericarp and then centrifuged for 10 min at 12000 rpm. Titration was continued using a 2,6‐dichlorophenolindophenol colored solution (3%) until the color of the extract changed to pale pink. The amount of vitamin C was calculated based on the following formula:

$$\text{Vitamin}\;C=\left(A/B\right)\times 100$$

In this equation, A is the standard concentration (mg mL^−1^) × titer value of the sample (mL) × 10 and B is the titer value of standard (mL) × sample volume (mL) × sample weight (mg). The amount of vitamin C was expressed in mg 100 g^−1^ DW.

### 2.6 Anthocyanin Content

Utilizing the pH difference method, the total anthocyanin content in rosehip pericarp extracts was assessed (Tabaszewska et al. 2020). Initially, 200 mL of desiccated pericarp was pulverized, and 5 mL of methanol/hydrochloric acid (1:1 v/v, pH = 2) was combined with it. Subsequently, the mixture of 4 mL buffer solution (pH = 1, prepared using HCl and sodium acetate) and 1 mL extract (pH = 4.5, prepared using HCl and KCl) was prepared, and the absorbance at 525 and 700 nm was measured using a spectrophotometer. The total anthocyanin content was expressed as mg of cyanidin 3‐glucoside per 100 g of dry weight (mg C3G 100 g^−1^ DW).

### 2.7 Total Carotenoid Content

The total carotenoid content was determined by following the method outlined by Ghazghazi et al. (2010). Pericarp powder was mixed with a solution of acetone, methanol, and petroleum ether in a 3:2:1 ratio, left at room temperature in the dark for 5 h, filtered using Whatman filter paper (No. 1), separated with diethyl ether, and dried in a rotary evaporator at 35°C. The resulting dry extract was dissolved in ethanol, combined with 60% potassium hydroxide, boiled for 10 min, partitioned with diethyl ether, evaporated, and dissolved in ethanol. The absorbance at 470 nm was measured using a spectrophotometer, and results were reported as mg β‐carotene equivalents per 100 g of dry weight (mg β‐CARE 100 g^−1^ DW).

### 2.8 Total Phenol and Flavonoid Content and Antioxidant Activity

The total phenol content (TPC) was determined by mixing 0.5 mL of methanolic extract with 5 mL of Folin–Ciocalteu solution (diluted 1:10 with distilled water) and then adding 4 mL of 1 M sodium carbonate. Instead of the extract, methanol was utilized as a control. The absorbance of the solutions was measured at a wavelength of 765 nm following a 30‐min incubation in the dark in a 40°C steam bath (Slinkard and Singleton 1977). The calibration curve was drawn with various concentrations of gallic acid standard (*R* ^2^ = 0.998, *y* = 0.01*x* + 0.0075).

For measuring the total flavonoid content (TFC), 0.5 mL of methanol extract was mixed with 1.5 mL of methanol, 0.1 mL of 10% aluminum chloride in ethanol (prepared from 10 g aluminum chloride in 100 mL ethanol), 2.8 mL of distilled water and 0.1 mL of 1 M potassium acetate. The control was prepared using pure methanol instead of a methanolic extract. The solutions absorbance was read after being exposed for 30 min in the dark at a wavelength of 415 nm (Chang et al. 2002) to determine the amount of total flavonoid from the quercetin standard curve and the equation of the line obtained from the standard curve (*R* ^2^ = 0.985, *y* = 0.0112*x* + 0.0004) was used. The percent inhibition method of DPPH decals was used to measure the antioxidant activity (Blois 1958). 2 mL DPPH (0.1 mmol) were mixed with 2 mL the prepared methanolic extract, and after 30 min of storage in the dark, the absorption of the samples was immediately read with a spectrophotometer at a wavelength of 517 nm. The control consisted of a combination of 2 mL DPPH and 2 mL methanol, and methanol served as the blank. The calculation for DPPH radical scavenging activity was determined using the equation provided below:

DPPH=Ac−As/Ac×100

In this equation, Ac is the DPPH radical absorption of the control sample and As is the DPPH absorption of the sample.

### 2.9 Determination of Antimicrobial Potential

The antifungal and antibacterial properties of *C. persica* fruit extracts were evaluated by determining the MIC values against model Gram‐positive ( *Staphylococcus aureus* ATCC12600) and Gram‐negative ( *Escherichia coli* ATCC11775) bacteria, as well as the yeast *Candida albicans* ATCC11006, which is a human pathogen. The minimum concentration required to inhibit visible growth of the microorganisms was established using the broth micro‐dilution method, following CLSI (Clinical Laboratory Standard Institute) guidelines. In summary, two‐fold serial dilutions of each extract were prepared in sterile plastic micro‐dilution trays using Mueller–Hinton broth, at concentrations ranging from 0.015 to 32 mg mL^−1^. Microbial suspensions for each bacterial and fungal strain were created from freshly cultured cells in sterile normal saline, adjusted to a turbidity of 0.5 McFarland standard. These suspensions were then added to the dilution trays and further diluted with sterile Mueller–Hinton broth (MHB) at a ratio of 1:100 for both yeast and bacteria. Consequently, the effects of each component concentration were analyzed on 0.5–1106 bacterial cells. The 96‐well plates were incubated at 37°C for 24 h, with resazurin used as a growth indicator. Each well received 4 μL of a 4 mg mL^−1^ stock solution of this reagent in sterile distilled water. Wells that turned pink indicated bacterial growth. Standard antibacterial agents, cefixime for bacteria and nystatin for yeast, were also tested as controls (Jorgensen and Turnidge 2015).

### 2.10 Data Analysis

Data variance analysis was done in a completely random design with 10 replications. Comparison of average data was conducted on the basis of LSD test at the *p* < 0.05 level using the SAS (Ver. 9.4) software. Excel 2016 software was used to draw graphs. In addition, cluster analysis, heat map, and correlation plot were drawn using R software and Origin software version 2024.

## 3 Results and Discussion

### 3.1 Morphological Characteristics

The results of the present study showed that there was significant variation among different ecotypes of *C. persica* in terms of fruit morphological and functional characteristics (Table 2). The highest coefficient of variation (CV %) was in pericarp weight (45.63%), fruit weight (36.91%), and seed weight (33.97%) traits. The high CV of the trait indicates the wide range of this trait quantitatively, which provides a wider range of selection for that trait for the breeder (Eghlima et al. 2023; Eghlima et al. 2024). Since the edible organ of this plant is the fruit, considering the high variation in fruit weight and pericarp weight, superior ecotypes in terms of these traits can be used for future breeding programs and achieve higher yielding ecotypes. The lowest (13.12 ± 1.24) and the highest (19.57 ± 1.29) fruit length to mm were recorded for the *CP4* and *CP5* ecotypes, respectively. The *CP4* ecotype exhibited the maximum fruit width (17.54 ± 2.14 mm), whereas the *CP1* ecotype showed the minimum fruit width (11.48 ± 1.03 mm). The minimum and maximum of fruit weights were 0.84 ± 0.08 and 2.53 ± 0.24 g related to *CP5* and *CP4* ecotypes, respectively. The amount of pericarps fruit varied between 0.21 ± 0.003 and 1.19 ± 0.044 g, the lowest and the highest of which was observed in ecotypes *CP1* and *CP3*, respectively. The heaviest seeds were 1.12 ± 0.14 g produced by the *CP4* ecotype and the lightest were 0.11 ± 0.02 g produced by the *CP5* ecotype. The minimum and maximum seed number per fruit were 1.00 ± 0.14 and 3.00 ± 0.45 related to *CP5* and *CP4* ecotypes, respectively. The results showed that there is a significant diversity in terms of morphological and functional traits. Previous studies have shown that there is a significant genetic diversity among wild fruits, which can be caused by the repeated reproduction of wild fruit seeds in natural habitats, which ultimately increases genetic diversity (Okatan et al. 2019, 2022; Guo et al. 2021).

**TABLE 2:** Comparison of different morphological traits among *Crataegus persica* ecotypes.

No.	Fruit length (mm)	Fruit width (mm)	Fruit weight (g)	Pericarp weight (g)	Seed weight (g)	Seed number per fruit
*CP1*	13.12 ± 1.24d	11.48 ± 1.03c	1.18 ± 0.14d	0.21 ± 0.003e	0.24 ± 0.03d	2.00 ± 0.16c
*CP2*	15.13 ± 1.04c	14.72 ± 1.36b	1.47 ± 0.11c	0.62 ± 0.027d	0.37 ± 0.09c	2.33 ± 0.29b
*CP3*	17.06 ± 1.18b	15.34 ± 1.51ab	1.83 ± 0.19b	1.19 ± 0.044a	0.45 ± 0.05b	2.33 ± 0.25b
*CP4*	19.57 ± 1.29a	17.54 ± 2.14a	2.53 ± 0.24a	0.69 ± 0.021c	1.12 ± 0.14a	3.00 ± 0.45a
*CP5*	12.05 ± 0.87d	12.10 ± 1.05c	0.84 ± 0.08e	1.13 ± 0.028b	0.11 ± 0.02e	1.00 ± 0.14d
CV (%)	22.51	17.31	36.91	45.63	33.97	12.57

*Note:* Data are mean ± standard deviation (*n* = 10). Values followed by the same letter within each column are significantly different (*p* < 0.05).

### 3.2 Phenolic Compounds

**FIGURE 2:**
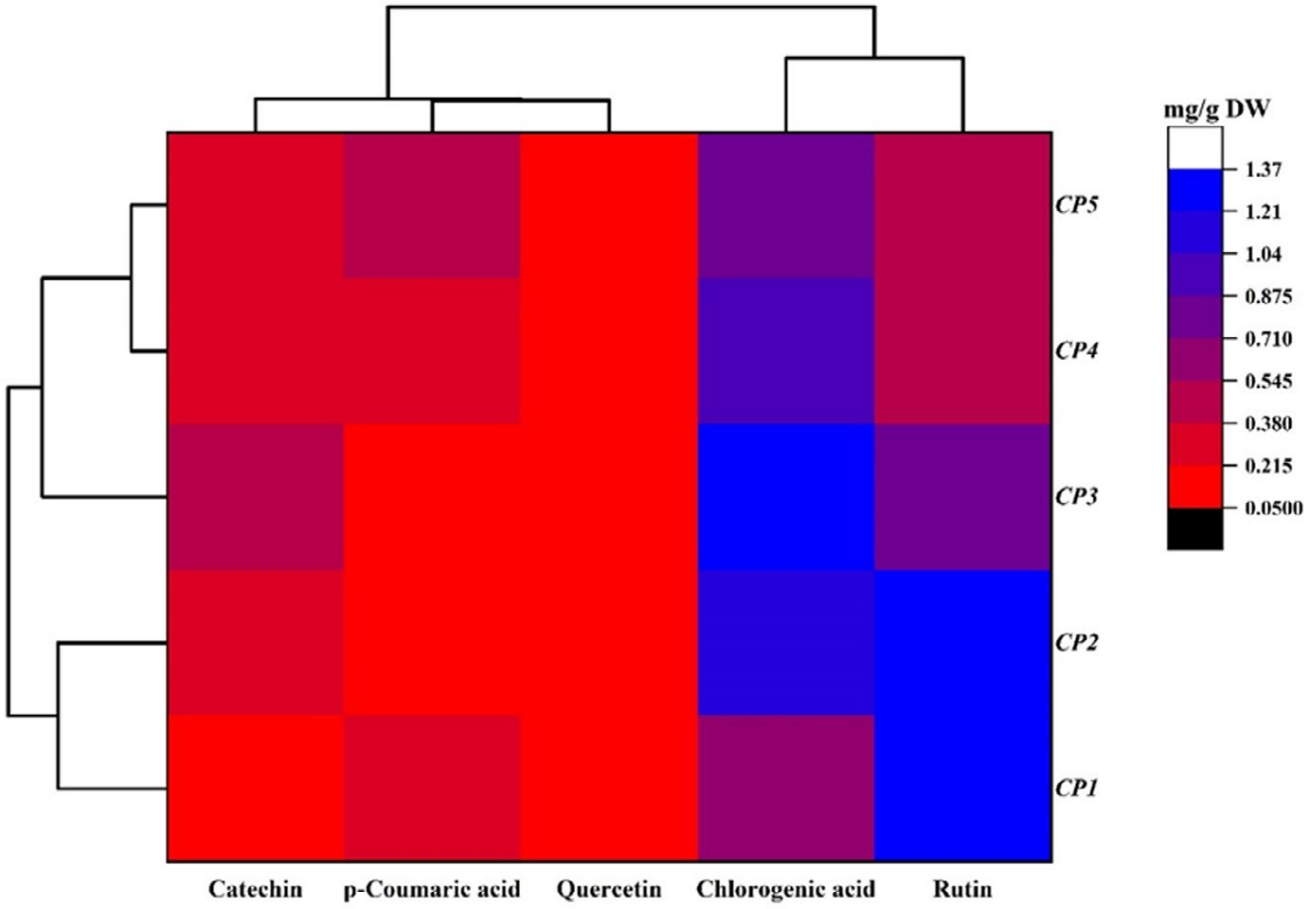
Heat map of the phenolic compound profiles of *Crataegus persica* ecotypes. Mean values refer to colors from minimum displayed in red to maximum represented with blue.

The main phenolic compounds in the *C. persica* included catechin, *p*‐coumaric, quercetin, chlorogenic acid, and rutin (Figure 2). The *CP3* ecotype had the highest level (0.47 mg g^−1^ DW) of catechin whereas the lowest level (0.17 mg g^−1^ DW) was observed in the *CP1* ecotype. The *p*‐coumaric and quercetin content varied from 0.11 (*CP3*) to 0.41 mg g^−1^ DW (*CP5*) and from 0.05 (*CP1*) to 0.17 mg g^−1^ DW (*CP3*), reflecting the high diversity of the studied ecotypes. Chlorogenic acid content was in the range of 0.65–1.37 mg g^−1^ DW. The lowest and the highest value of the chlorogenic acid were detected in *CP1* and *CP3* ecotypes, respectively. The *CP2* ecotype had the maximum level (1.31 mg g^−1^ DW) of rutin whereas the minimum level (0.54 mg g^−1^ DW) was observed in the *CP4* ecotype. Salmanian et al. (2014) identified the chlorogenic acid, caffeic acid, and gallic acid from pulp extracts of *C. pentagyna* . Study on the 56 *Crataegus* spp. genotypes of Iran showed that chlorogenic acid, hyperoxide and rutin were the major phenols in flowers extracts (Alirezalu et al. 2018). Pavlovic et al. (2019) reported chlorogenic acid, *p*‐coumaric acid, ferulic acid, quercetin, caffeic acid, and rutin as the most abundant phenolic compounds in *C. pentagyna* from Serbia. Rutin, vitexin, chlorogenic acid, hyperoxide, quercetin, and quercetin were the main phenolic compounds in 14 *Crataegus* species grown in Iran (Alirezalu et al. 2020). Variation in the phenolic compounds of different ecotypes can be caused by the influence of geographical and climatic conditions, genetics, and soil characteristics (Butkeviciute et al. 2022).

### 3.3 Biochemical Traits

The biochemical profile analysis of five *C. persica* ecotypes revealed significant intraspecific variation, offering valuable insights into the species' phytochemical diversity and potential adaptive strategies (Table 3). This observed variability not only indicates the importance of ecotype‐specific characterization but also presents intriguing implications for both evolutionary biology and potential nutraceutical applications. Total anthocyanin content exhibited a remarkable two‐fold range across ecotypes, with *CP2* demonstrating the highest concentration (85.34 ± 2.41 mg C3G g^−1^ DW) and *CP1* the lowest (43.21 ± 1.27 mg C3G g^−1^ DW). This substantial variation suggests differential regulation of anthocyanin biosynthesis pathways among ecotypes, potentially reflecting adaptations to varying environmental stressors or pollinator preferences. The observed anthocyanin gradient (*CP1* < *CP5* < *CP4* < *CP3* < *CP2*) merits further investigation to elucidate the underlying genetic and environmental factors driving this pattern. Altitude is one of the most important factors affecting the growth and biochemical traits of plants as along with the increase in altitude, the amount of radiation and the quality of sunlight and the difference between day and night temperature have increased and then respiration rate will be decreased as a result of decreased temperature of night. So the amount of photosynthesis will be accompanied by a decrease in the intensity of respiration at night, and the increase in photosynthesis will increase the amount of photoassimilates, which is a protective factor against cold stress in colder conditions, so that the plant can partially compensate the damage caused by the decrease in photosynthesis (Cirak et al. 2017). It has also been proven that the amount of UV‐B rays increases with the increase in altitude, which causes overexpression of oxidase and superoxide dismutase genes, so that the amount of anthocyanin accumulates more to compensate for the harmful effects of ultraviolet rays (Khoo et al. 2017). TSS and total soluble carbohydrates (TSC) displayed considerable variability, ranging from 10.31°Brix ± 0.62°Brix to 18.53°Brix ± 0.97°Brix and 8.34% ± 0.46% to 16.56% ± 1.21%, respectively. Notably, *CP1* exhibited maximum TSS and TSC content, contrasting with *CP3* and *CP4*, which showed the lowest values for these parameters. This diversity in sugar content aligns with previous findings in Turkish *Crataegus* species (Gundogdu et al. 2014), suggesting a conserved pattern of variability within the genus. The observed differences may reflect varying osmotic regulation strategies or differences in carbon allocation among ecotypes. Carotenoid content varied substantially, with *CP2* exhibiting the highest concentration (294.4 μg g^−1^ DW) and *CP5* the lowest (185.32 μg g^−1^ DW). This 1.6‐fold difference in carotenoid levels could be indicative of divergent photoprotective strategies or variations in fruit maturation processes among ecotypes. The findings of Zheng et al. (2023) regarding high‐altitude adaptations in Tibetan peach fruit carotenoids provide an intriguing parallel, suggesting that altitudinal gradients may play a crucial role in shaping carotenoid profiles in *C. persica* as well. Vitamin C content demonstrated a nearly three‐fold range, from 12.38 mg g^−1^ DW in *CP1* to 4.24 mg g^−1^ DW in *CP4*. Interestingly, *CP1*, collected from the highest altitude (1681 m) in West Azerbaijan‐Piranshahr (Table 1), exhibited the highest vitamin C concentration. This observation aligns with previous studies suggesting that high‐altitude environments may stimulate increased ascorbic acid synthesis as an adaptive response to elevated oxidative stress (Zoratti et al. 2015). The significant variability observed in biochemical traits across *C. persica* ecotypes can be attributed to a complex interplay of genetic factors, environmental conditions (including weather and climate), and soil characteristics (Gundogdu et al. 2014). The altitude‐dependent trends observed in some parameters, particularly vitamin C and potentially carotenoids, suggest that elevational gradients may be a key driver of phytochemical diversity in this species. Although the genotype of the plants is the main factor that determines the concentration of vitamin C among berries, levels of ascorbic acid are also influenced by different environmental factors, such as solar light and altitude as demonstrated in our results also. In this case, Kumar et al. (2019) demonstrated that vitamin C content was more in the apples harvested from higher altitudes (1800 m) than in the lower altitudes (1400 m). Ascorbic acid is made from carbohydrate with higher light intensity and increased photosynthesis over time and accumulates in the plant, which shows that high concentration of ascorbic acid from fruits grown at high altitudes had more light intensity than the fruits grown at lower altitudes, it was directly related to less‐received light intensity. They also stated that lower average temperature at higher altitudes is another factor of vitamin C accumulation in the plant and also fruits. The observed biochemical diversity may reflect adaptive radiation within *C. persica* , enabling the species to thrive across varied ecological niches. Further research into the genetic basis of this variability could provide insights into the evolutionary history and adaptive potential of the species. The significant variations in bioactive compounds, particularly anthocyanins, carotenoids, and vitamin C, highlight the potential for targeted selection and breeding programs aimed at developing *C. persica* cultivars with enhanced nutritional profiles. Given the apparent influence of environmental factors on phytochemical composition, these results underscore the potential vulnerability of *C. persica* populations to climate change. Monitoring biochemical profiles across ecotypes over time could serve as a sensitive indicator of ecosystem responses to environmental shifts. The unique biochemical signatures of different ecotypes emphasize the importance of preserving diverse *C. persica* populations to maintain the species' overall genetic and phytochemical diversity. Furthermore, as shown different amounts of vitamin C between different *Crataegus* species (Gundogdu et al. 2014), the same variation was observed among five ecotypes, although fruits collected from the highest altitude (*CP1*) contain the most amount of vitamin C. It seems that carotenoid content is mainly affected by the environmental factors, light, and temperature as well as altitude so that the results of Zheng et al. (2023) showed that the abundant variation in carotenoids was highly associated with high‐altitude adaptations in Tibetan peach fruit. Also, higher altitudes, decrease in temperature and relative humidity, as well as increase in light quality are the most important reasons that stimulate the synthesis mechanism of photosynthetic pigments and phenolic compounds.

**TABLE 3:** Comparison of different biochemical traits among *Crataegus persica* ecotypes.

Traits	*CP1*	*CP2*	*CP3*	*CP4*	*CP5*
Total anthocyanins content (mgC3G 100 g^−1^DW)	43.21 ± 1.27e	85.34 ± 2.41a	75.34 ± 1.94b	59.11 ± 1.13c	47.78 ± 1.26d
Total soluble solids (°Brix)	13.43 ± 0.81a	18.53 ± 0.97a	10.31 ± 0.62d	14.28 ± 0.84b	11.61 ± 0.32c
Total soluble carbohydrate (%)	16.56 ± 1.21a	11.54 ± 0.84c	13.45 ± 1.05b	8.34 ± 0.46d	14.75 ± 1.27b
Total carotenoid content (μg g^−1^ DW)	271.32 ± 3.38b	294.4 ± 2.74a	192.43 ± 1.83d	256.61 ± 2.42c	185.32 ± 1.63e
Vitamin C (mg 100 g^−1^ DW)	12.38 ± 0.86a	6.75 ± 0.43c	10.72 ± 0.94b	4.24 ± 0.34e	5.12 ± 0.38d

*Note:* Data are mean ± standard deviation (*n* = 10). Values followed by the same letter within each row are significantly different (*p* < 0.05).

### 3.4 Total Phenol and Flavonoid Content and Antioxidant Activity

**FIGURE 3:**
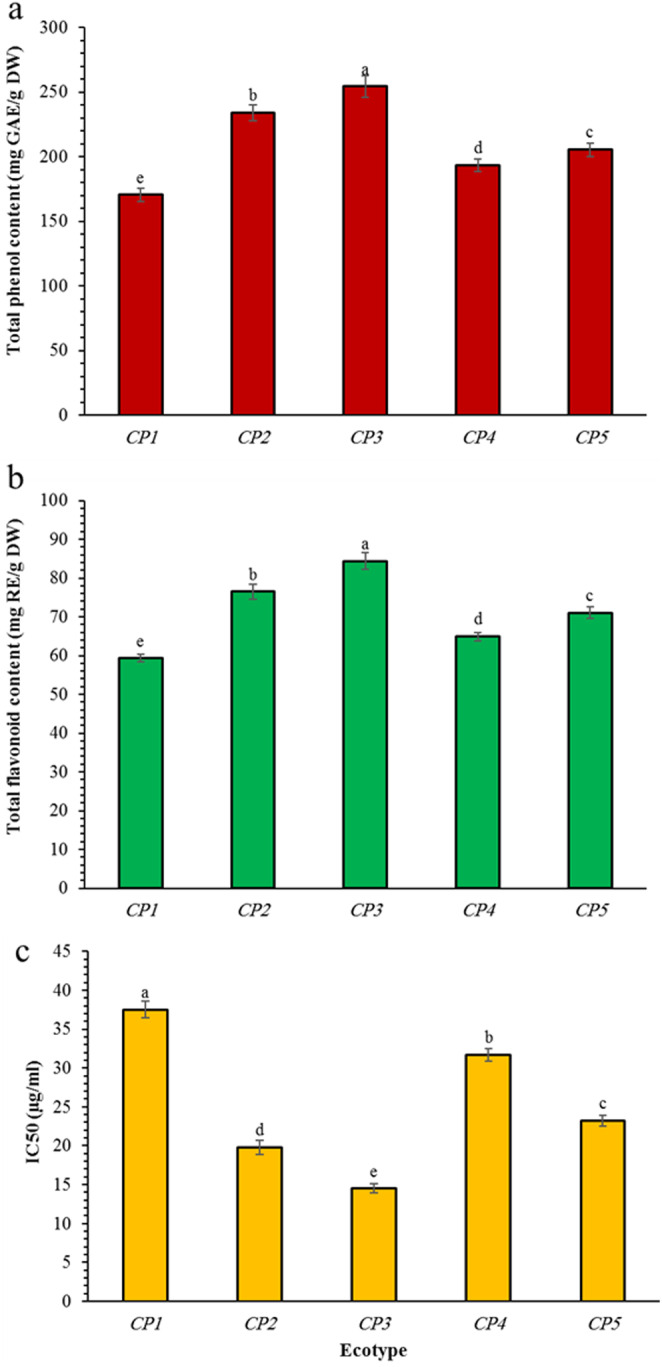
Histogram of total phenol content (a), total flavonoid content (b), and antioxidant activity (c) among *Crataegus persica* ecotypes. *Different letters indicate significant differences based on LSD test analysis at *p* ≤ 0.05. Data are mean values ± SE.

The wide variability of total phenol, flavonoid and antioxidant activity was observed among ecotypes (Figure 3). The same behavior was detected in total phenol and flavonoid content among different ecotypes as total phenol and flavonoid content of the fruit extracts of different ecotypes increased in the order of *CP1* < *CP4* < *CP5* < *CP2* < *CP3*. The maximum and minimum number of total phenols and flavonoids was recorded respectively in fruits of *CP3* and *CP1* (Figure 3). In general, total content of phenols was higher than of flavonoids in fruit extracts of all *Crataegus* ecotypes as illustrated in Figure 3a,b. The same trend was also demonstrated in leaf extracts of the other species of *Crataegus* as suggested in the other literatures (Dekić et al. 2020). The difference in flavonoid content is likely due to the presence of procyanidins or other phenolic acids as described previously by Cui et al. (2006) and Liu et al. (2011) in fruits of *C. pinnatifida* . On the other hand, the environmental factors influenced by specially altitude seems to be important in phenolic and flavonoid compounds content among tested ecotypes, although some researchers stated that the genetic origin is more important (Bignami et al. 2001). Regarding the antioxidant activity, the lower IC_50_ values correspond to the stronger antioxidant activity of the extracts. So, the same as similar pattern of total phenols and flavonoids, there was a relationship between the mentioned traits and the antioxidant activity of *C. persica* fruits (Figure 3c) as the maximum and minimum amount of antioxidant activity was the same with those shown in total phenols and flavonoids, that is, *CP3* had the maximum (37.5 μg mL^−1^) antioxidant activity whereas *CP1* showed the minimum (14 μg mL^−1^) activity (Figure 3c). Despite the direct relationship between phenols and flavonoid content with antioxidant capacity of the fruit as previously suggested by Dekić et al. (2020), it seems that having the maximum pericarp weight (Table 2) can be influenced on the content of phenols and flavonoids compared with the other ecotypes. *CP3* ecotype which is collected from West Azerbaijan‐Sardasht had the minimum altitude (1205 m) compared with the other habitats. However, the higher amounts of phenols and flavonoids as well as higher capacity for scavenging free radicals (assayed by DPPH) could be attributed to the lower altitude in plants habitat which is depicted also in other researcher findings (Guerrero‐Chavez et al. 2015). Our analysis of *C. persica* fruit ecotypes reveals a complex and intriguing landscape of phytochemical variation that extends beyond mere quantitative differences. The systematic variations in phenolic and flavonoid content across different ecotypes not only highlight the remarkable biochemical plasticity of this genus but also underscore the profound influence of environmental factors, particularly altitude, on plant secondary metabolite production. The *CP3* ecotype, originating from West Azerbaijan‐Sardasht at the lowest altitude (1205 m), demonstrated the most remarkable phytochemical profile, exhibiting the highest total phenol and flavonoid content alongside the strongest antioxidant activity. These finding challenges simplistic linear assumptions about plant metabolite accumulation and suggests a nuanced interplay between geographical origin, physiological adaptation, and biochemical potential. The observed positive correlation between lower altitude and enhanced phytochemical concentration suggests that ecological context plays a critical role in modulating plant chemical defense mechanisms. Furthermore, the substantial variability among ecotypes emphasizes the importance of local germplasm preservation and targeted ecological research, as each geographical variant represents a unique biochemical repository with potential pharmaceutical and nutritional significance. These results not only contribute to our understanding of *Crataegus persica*'s chemical diversity but also open promising avenues for future research in plant biochemistry, ecological adaptation, and potential therapeutic applications.

### 3.5 Correlation, Cluster, and Factor Analysis

**FIGURE 4:**
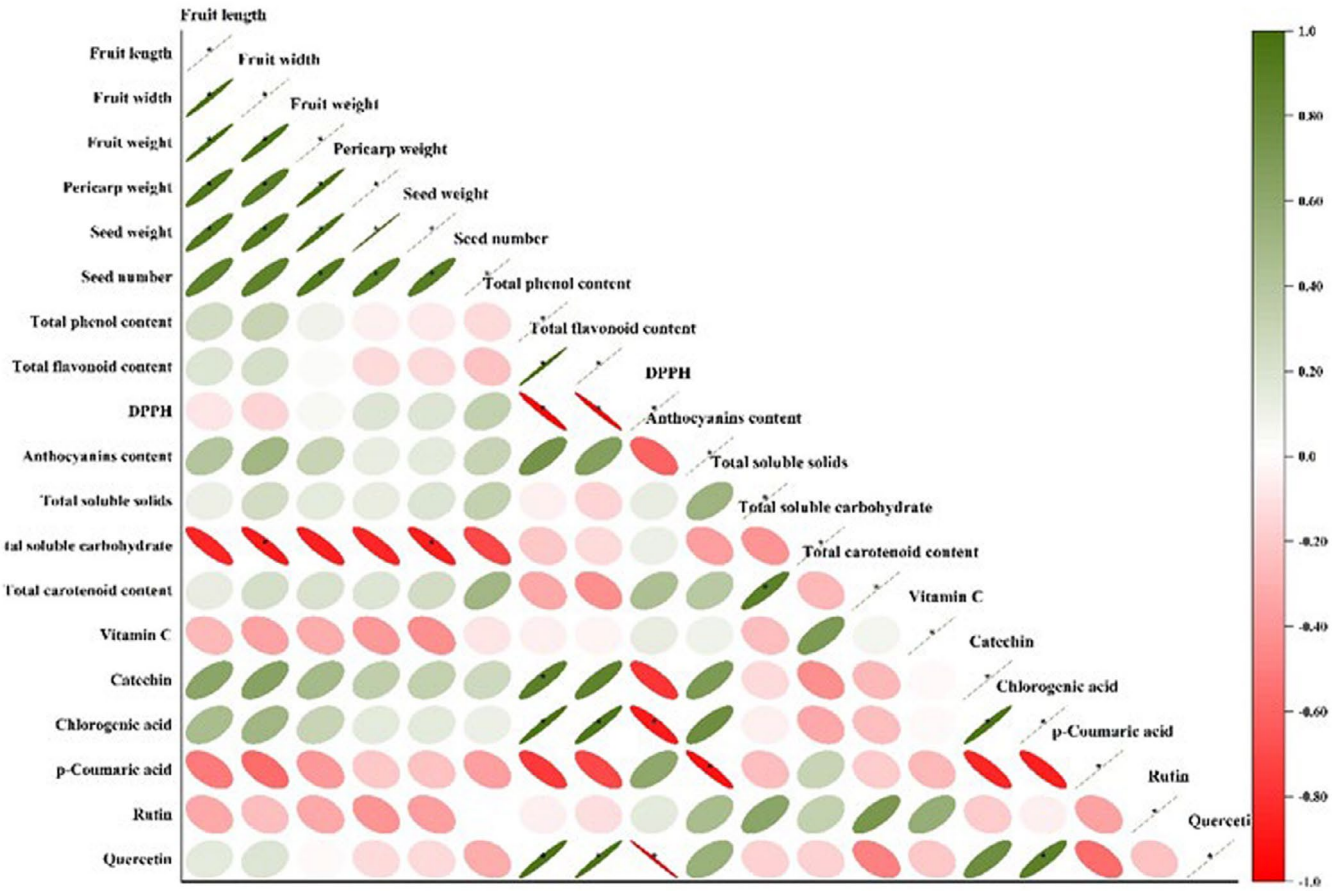
Linear correlation between the morphological and phytochemical traits. Significant difference in 5% level.

**FIGURE 5:**
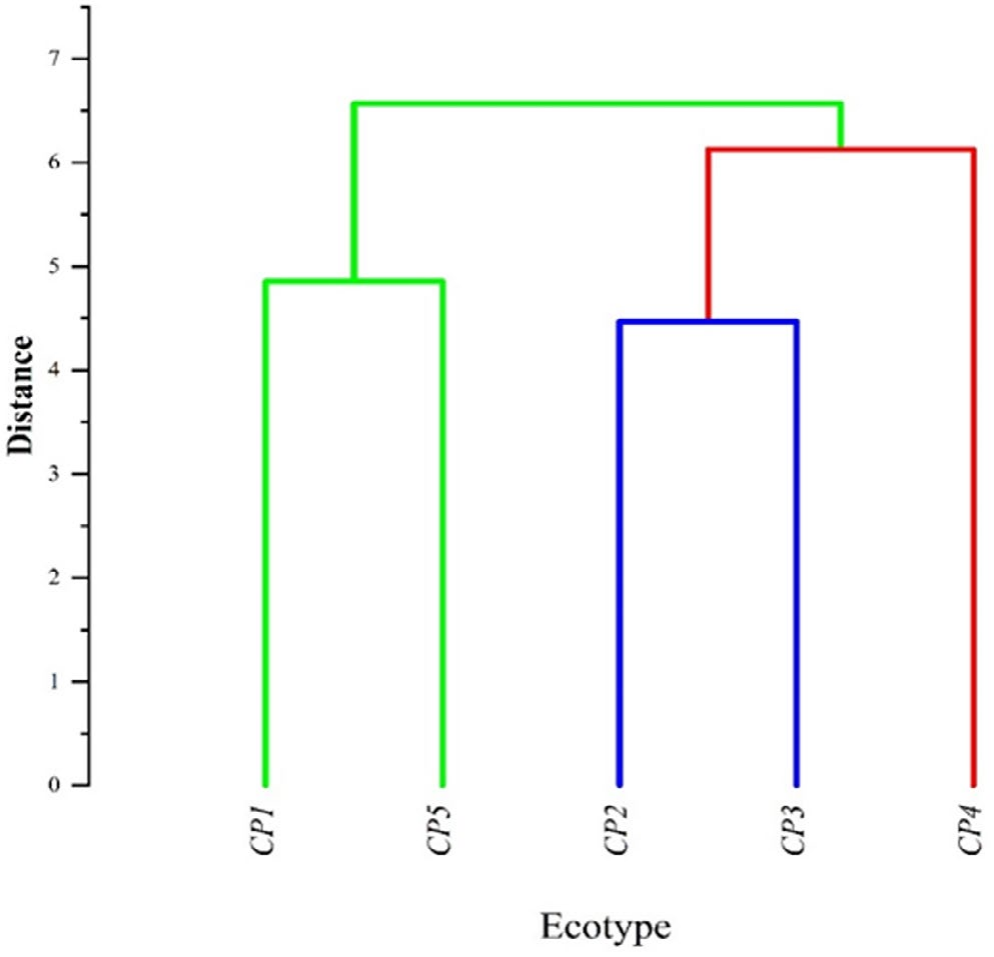
Ward cluster analysis of *Crataegus persica* ecotypes based on morphological and phytochemical traits using Euclidean distances.

**FIGURE 6:**
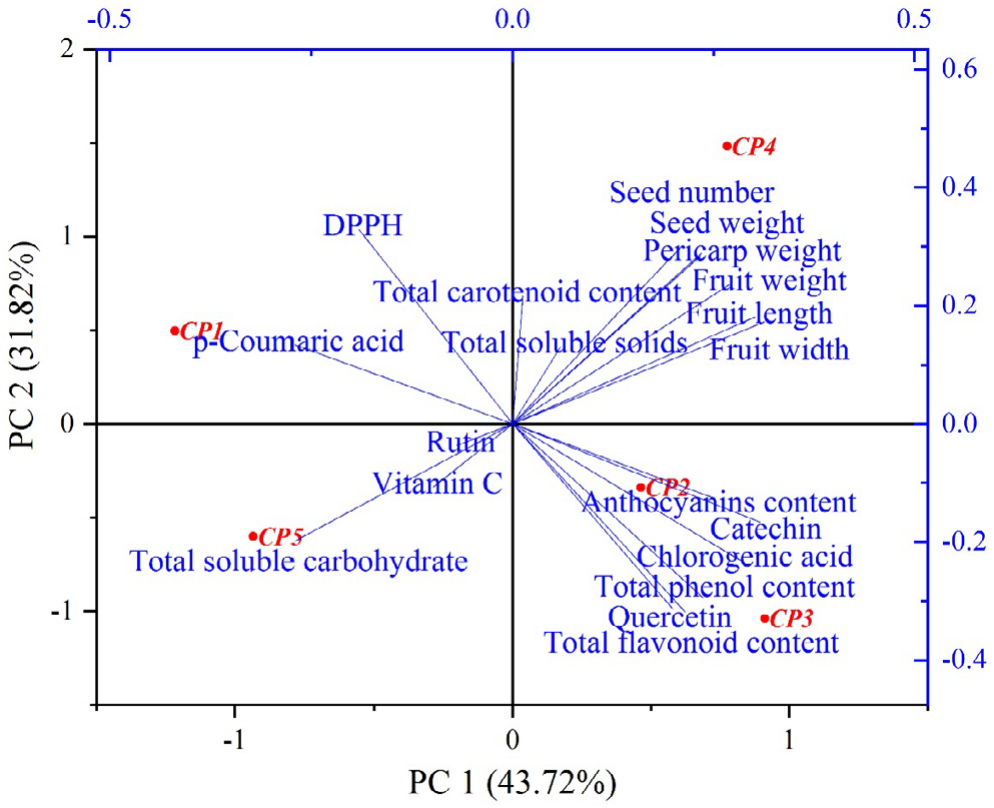
Biplot analysis of *Crataegus persica* ecotypes based on phytochemical and morphological traits.

The relationships between morphological and phytochemical features were calculated by Pearson's correlation and displayed by network and heat map (Figure 4). Fruit length had positive and significant correlations with fruit width, fruit weight, and pericarp weight, and seed number per plant had positive and significant correlations with fruit and pericarp weight. The TPC was positively and significantly correlated with the TFC, catechin, chlorogenic acid, and quercetin and negatively correlated with IC_50_. In addition, TSC showed negative and significant correlation with fruit width and seed weight. Having sufficient knowledge of the relationships between traits in plants is essential for selection and breeding programs (Eghlima et al. 2020). The results of the cluster analysis showed that the ecotypes were placed in three main groups (Figure 5). Ecotype *CP4*, which was placed in the first group, was superior to other ecotypes in terms of fruit length and width, fruit weight, pericarp weight, seed weight, and number of seeds in the fruit. Ecotypes *CP2* and *CP3* were superior in terms of total phenol content, total flavonoid content, antioxidant activity, anthocyanin content, TSS, total carotenoid content, catechin, and chlorogenic acid traits and were placed in the second group. Ecotypes *CP1* and *CP5* were placed in the third group, which had the highest total soluble carbohydrate. The grouping of the ecotypes was relatively unrelated to their geographical distribution, and the distribution of individuals in the cluster did not follow a specific geographical pattern, which was similar to the results of other studies on *G. glabra* (Ahmadi Hosseini et al. 2014), *Satureja khuzestanica* (Hadian et al. 2011), *Anemopsis californica* (Medina‐Holguin et al. 2007), which reported that the difference in chemical composition due to different genetic and environmental determinants. Variation in the plant phytochemicals depends on both genetic and environmental factors, although the influence of genetic factor has more predictable. Contribution of different genes including phenylalanine ammonia lyase (PAL), cinnamic acid 4‐hydroxylase (C4H), hydroxycinnamate coenzyme A ligase (4CL), tyrosine aminotransferase (TAT), 4‐hydroxyphenylpyruvate reductase (HPPR), chalcone synthase (CHS), chalcone reductase (CHR), chalcone isomerase (CHI), and flavonol synthase (FLS) in the biosynthesis and accumulation of phenolics and flavonoids varied in different plant genotypes (Selseleh et al. 2020). The influence of climatic and geographical conditions on gene expression levels, as well as the diversity of morphological traits and phytochemical compounds among plant populations, is known. Diversity among plants of a species is caused by environmental factors and the structure of genes, which is well shown in cluster analysis when similar populations are placed close to each other (Pourhosseini et al. 2020). Based on the fourth PCA results, the main factor explained a total of 97.99% of the total variance between ecotypes (Table 4). The first, second, third, and fourth factors accounted for 73.43%, 82.31%, 79.17%, and 66.5% of the total variance, respectively. Fruit length, fruit width, fruit weight, anthocyanins content, total soluble carbohydrate, catechin, chlorogenic acid, and *p*‐coumaric acid were the traits in the first component that had the highest impact factors. The greatest impact factors in the second component were related to pericarp weight, seed weight, seed number, total phenol content, total flavonoid content, antioxidant activity, and quercetin. This analysis can clarify the main differentiating factors between the studied populations. In Eghlima et al.'s (2024) study, PCA was used to evaluate different ecotypes of *Equisetum arvense* , and the studied agro‐morphological traits were categorized into three main components, which accounted for 96.77% of the total variance. In another study on *Rosa canina* , phytochemical and morphological characteristics were categorized into seven principal components using PCA, accounting for 93.53% of the total variance (Bakhtiar et al. 2023). The biplot also confirms the results of the cluster analysis and ecotypes close to each other were placed in one group (Figure 6). On the other hand, the selection of morphological and phytochemical traits in this study was guided by their potential relevance to plant adaptation and genetic diversity. Although climatic, soil, and light‐related factors were not directly measured, their indirect influence is reflected in the observed variations among ecotypes. The diverse morphological and phytochemical characteristics suggest complex interactions between genetic potential and environmental conditions. The significant correlations between traits, such as fruit dimensions and phytochemical content, indicate potential physiological trade‐offs and adaptive strategies developed by these plant populations in response to their specific geographical contexts. The cluster and principal component analyses revealed distinct groupings that potentially represent localized adaptations, highlighting the importance of considering both genetic and environmental factors in understanding plant diversity. However, future research could benefit from integrating more detailed environmental data, including precise geographical coordinates, soil composition, and microclimate parameters, to establish more comprehensive relationships between plant traits and their ecological contexts. By expanding the analytical approach to include these additional environmental parameters, researchers can develop a more nuanced understanding of how different ecotypes respond to and are shaped by their specific environmental conditions.

**TABLE 4:** Eigenvalues of the principal component axes from the PCA of the studied traits in *Crataegus persica* ecotypes.

Traits	Component			
	I	II	III	IV
Fruit length	0.869^a^	0.445	−0.126	0.174
Fruit width	0.904^a^	0.424	−0.025	0.044
Fruit weight	0.787^a^	0.587^a^	−0.124	0.146
Pericarp weight	0.674^a^	0.699^a^	−0.219	0.095
Seed weight	0.678^a^	0.711^a^	−0.183	0.047
Seed number	0.604^a^	0.737^a^	0.175	0.249
Total phenol content	0.685^a^	−0.725^a^	0.034	−0.068
Total flavonoid content	0.619^a^	−0.783^a^	−0.046	−0.043
DPPH	−0.550^a^	0.810^a^	0.092	0.182
Anthocyanins content	0.723^a^	−0.309	0.615^a^	−0.066
Total soluble solids	0.171	0.322	0.747^a^	−0.555^a^
Total soluble carbohydrate	−0.781^a^	−0.488	0.112	0.373
Total carotenoid content	0.037	0.534^a^	0.826^a^	−0.177
Vitamin C	−0.284	−0.263	0.444	0.808^a^
Catechin	0.888^a^	−0.409	−0.057	0.204
Chlorogenic acid	0.818^a^	−0.569^a^	0.051	0.066
*p*‐Coumaric acid	−0.780^a^	0.329	−0.472	−0.244
Rutin	−0.201	−0.090	0.973^a^	0.070
Quercetin	0.575^a^	−0.771^a^	−0.176	−0.207
Eigenvalue	8.30	6.04	3.19	1.45
% of variance	43.72	31.82	16.79	5.66
Cumulative %	43.72	75.54	92.33	97.99

^a^ Eigenvalues significant > 0.50.

### 3.6 Antimicrobial Potential

Three kinds of harmful human pathogens ( *Staphylococcus aureus* , *Escherichia coli* , and *Candida albicans* ) were used to assess the antibacterial properties of *C. persica* fruit extracts. Generally, among all ecotypes, the minimum antimicrobial effect of the extract was observed against *E. coli* with average 12.8 MIC, compared to the other pathogens, although between *S. aureus* and *C. albicans* the antibacterial inhibitory effect was more potent in *S. aureus* (as 1.175 average MIC) against *C. albicans* with up to 2.05 average MIC (Table 5). Ojagh et al. (2010) suggested that compared with the Gram‐negatives, Gram‐positive bacteria are generally more sensitive to plant extracts, and the plant extracts have more lethal effects than inhibitory effects against bacteria. For the effective inhibitory effect of *C. persica* on other bacteria, especially *E. coli* , the higher concentrations of the mentioned extract should be used. Also, according to the deference among all ecotypes, *CP2* and *CP3* could be more effective than the others. On the other hand, the highest antimicrobial effect was recorded in ecotype *CP3* followed by ecotype *CP2* against to *S. aureus* (Table 5).

**TABLE 5:** Antimicrobial activities of different ecotypes of *Crataegus persica*.

Ecotype	Minimum inhibitory concentration (MIC mg mL^−1^)		
	*Staphylococcus aureus*	*Escherichia coli*	*Candida albicans*
*CP1*	2.50	16	0.25
*CP2*	0.50	8	1
*CP3*	0.125	8	1
*CP4*	1.25	16	4
*CP5*	1.50	16	4

## 4 Conclusions

The high nutritional quality of the studied fruit demonstrated the importance of the mentioned fruit in human health as a bioactive compounds source. The study revealed that there were differences in terms of fruit characteristics among hawthorn species, and thus, better quality hawthorn genotypes can be selected within the species. Hence, this study is considered to be a valuable reference for future studies. Among all ecotypes, fruits of ecotype *CP1* and *CP2* had the better quality because of the highest amount of biochemical traits such as TSS, TSC, vitamin C in *CP1* and total anthocyanin, TSS, and total carotenoid in *CP2*. Ecotypes *CP1* and *CP2* was related to the fruits which are collected respectively from West Azerbaijan‐Piranshahr and Mahabad with respectively 1681 and 1485 m altitude. The results suggested the high potential of health benefits and bioactive compounds of *CP1* and *CP2* ecotypes. On the other hand, *CP1* ecotype had the maximum antioxidant activity, although the morphological traits of the fruits were not so impressive. However, the highest amount of total phenols and flavonoids were related to the *CP3* ecotype with the maximum pericarp weight. Evaluation of the antibacterial effects of the *C. persica* extracts also revealed that *CP3* ecotype was more potent in coping on the bacteria specially *Staphylococcus aureus* at 0.125 minimum inhibitory concentration which is due to the high capacity of total phenols, flavonoids and antioxidant capacity. Generally, the observed morphological, biochemical and antimicrobial characteristics among different *C. persica* ecotypes can be influenced by altitude which is associated to general climatic trends, including reduced temperature, increased radiation, decreased atmospheric pressure and other environmental changes.

## Author Contributions

**Ghasem Eghlima:** methodology, sample collection, conceptualization, supervision, data curation, data analysis and writing‐original draft. **Fateme Aghamir:** lab work, analysis data. **Meisam Mohammadi:** methodology, conceptualization, data curation, reviewing, and editing. **Hanifeh Seyed Hajizadeh:** writing – original draft (equal). **Hanifeh Seyed Hajizadeh and Ozkan Kaya:** conceptualization (equal), writing – original draft (equal), writing – review and editing (equal).

## Ethics Statement

The authors have nothing to report.

## Consent

The authors have nothing to report.

## Conflicts of Interest

The authors declare no conflicts of interest.

## Data Availability

Correspondence and requests for materials should be addressed to Ghasem Eghlima.
